# Performance of statistical methods on CHARGE targeted sequencing data

**DOI:** 10.1186/s12863-014-0104-9

**Published:** 2014-10-03

**Authors:** Chuanhua Xing, Josée Dupuis, L Adrienne Cupples

**Affiliations:** Department of Biostatistics, Boston University, Boston, MA USA; Framingham Heart Study, Framingham, MA USA

**Keywords:** Case-cohort design, CHARGE targeted sequencing data, Rare variants, Type I error, Power, SKAT, Score-Seq, Madsen and browning, Burden tests

## Abstract

**Background:**

The CHARGE (Cohorts for Heart and Aging Research in Genomic Epidemiology) Sequencing Project is a national, collaborative effort from 3 studies: Framingham Heart Study (FHS), Cardiovascular Health Study (CHS), and Atherosclerosis Risk in Communities (ARIC). It uses a case-cohort design, whereby a random sample of study participants is enriched with participants in extremes of traits. Although statistical methods are available to investigate the role of rare variants, few have evaluated their performance in a case-cohort design.

**Results:**

We evaluate several methods, including the sequence kernel association test (SKAT), Score-Seq, and weighted (Madsen and Browning) and unweighted burden tests. Using genotypes from the CHARGE targeted-sequencing project for FHS (n = 1096), we simulate phenotypes in a large population for 11 correlated traits and then sample individuals to mimic the CHARGE Sequencing study design. We evaluate type I error and power for 77 targeted regions.

**Conclusions:**

We provide some guidelines on the performance of these aggregate-based tests to detect associations with rare variants when applied to case-cohort study designs, using CHARGE targeted sequencing data. Type I error is conservative when we consider variants with minor allele frequency (MAF) < 1%. Power is generally low, although it is relatively larger for Score-Seq. Greater numbers of causal variants and a greater proportion of variance improve the power, but it tends to be lower in the presence of bi-directionality of effects of causal genotypes, especially for Score-Seq.

**Electronic supplementary material:**

The online version of this article (doi:10.1186/s12863-014-0104-9) contains supplementary material, which is available to authorized users.

## Background

Genome-wide association studies (GWAS) have identified hundreds of disease susceptible loci that harbor common variants, but most are not causal and explain only a small portion of the genetic risk for most diseases. The role of rare variants with minor allele frequency (MAF) < 0.05 has not been comprehensively explored in GWAS, while rare variant associations are believed to play an important role in disease etiology [1-12]. Emerging sequencing technologies allow for the characterization of virtually all of an individual’s genetic variation. Hence, motivations for this work are: 1) the shift in measurement of genetic variants away from common variation using genotyping arrays to genotyping or sequencing of rare variants, requiring greater understanding of rare variant methods; and 2) the high cost of sequencing requires careful consideration of efficient study designs. Here we discuss the case-cohort study design for sequencing studies and evaluate the possible limitations of current methods for data collected under this study design.

The Cohorts for Heart and Aging Research in Genomic Epidemiology (CHARGE) Sequencing Project is a national, collaborative effort from three studies: Framingham Heart Study (FHS), Cardiovascular Health Study (CHS) and Atherosclerosis Risk in Communities (ARIC). What makes the CHARGE sequencing study different from other studies is its case-cohort design, where a cohort random sample plus selected individuals with extreme values from one or more pre-specified traits are considered for analysis. Such a study design is advantageous when investigators wish to examine multiple traits. One component of the CHARGE targeted sequencing study involves 1096 individuals from FHS, consisting of a cohort random sample of 504 study participants from the Offspring Cohort and 592 participants selected from the extremes of 11 traits.

In recent years, many statistical approaches have been developed to jointly analyze multiple rare variants in aggregate-based tests to gain power. But current statistical methods for rare variant association studies rarely consider a case-cohort design, and hence potential bias in estimation and type-I error might be observed in analyses of CHARGE targeted sequencing data. The methods developed to date are generally for studies in which participants are assumed to be independent and a random representation of the general population such as case–control design. Typically, all study participants are considered for analyses in a case-cohort design; for dichotomous traits, participants affected by a specific disease or trait are considered as cases and other participants carrying other diseases or from a random non-diseased sample are considered as controls; for quantitative traits, all participants are used in genetic association studies of a specific disease trait, but potential bias in effect estimates may arise when including selected extreme values. Some participants as “potential risk carriers” for multiple traits can also make the issue even more complex. The uniquely ascertained participants in a case-cohort design with correlated traits form a non-representative dataset and may generate biases.

To address the concerns regarding the case-cohort study design and application of methods for rare variants, we evaluate type I error and power of statistical methods for aggregate-based association tests of rare variants in the case-cohort study design of the CHARGE targeted sequencing project. We examine the statistical performance of commonly used methods, the Sequence Kernel Association Test (SKAT) [13], Score-Seq [14], weighted [15], and unweighted (T1 [16]) burden tests. These methods have been well-studied using simulated data. Although Ladouceur et al., [17] used Sanger sequencing from 1998 individuals for both continuous and binary traits in their power comparison, they did not perform type 1 error comparisons. Our work contributes in the following aspects. (1) We evaluate the statistical performance of several statistical methods that aggregate data in a genomic region on measured CHARGE targeted sequencing data based on a case-cohort design. (2) We evaluate over seventy-seven targeted sequencing regions in CHARGE, representing a wide range of genotype structures. (3) We consider complex, correlated phenotypes. (4) We evaluate both type I error and power, because power is a valid measure only if type I error is properly controlled. We aim to provide some guidelines on the performance of these methods to detect associations with rare variants on CHARGE targeted sequencing data using the case-cohort study design.

## Methods

### Data

We used genotypes from our CHARGE targeted-sequencing project for the Framingham Heart Study (n = 1096), and we simulated correlated phenotypes to mimic the potential relationship among phenotypes in real data. We generated 11 correlated traits similar to those found in FHS for a very large population of 12,000 people, using the method described by Lumley et al. (http://stattech.wordpress.fos.auckland.ac.nz/files/2012/05/design-paper.pdf). The correlation between traits was induced by an iterative process. The initial trait was generated using a *t* distribution with 15 degrees of freedom. The number of traits was doubled after each iteration; half were generated by adding the previous traits to a randomly generated *t* value, and the second half were generated by adding the negative of the previous traits to a randomly generated *t* value. We generated 2^4^ = 16 traits in this manner, and we selected the first 11 traits for analysis. The correlation among the traits is given in Table 1.

**Table 1:** **Correlation among 11 traits**

**Traits\Traits**	**1**	**2**	**3**	**4**	**5**	**6**	**7**	**8**	**9**	**10**	**11**
**1**	1	0.6	0.2	0.6	−0.2	0.2	0.6	0.2	−0.6	−0.2	0.2
**2**	0.6	1	0.6	0.2	0.2	−0.2	0.2	0.6	−0.2	−0.6	−0.2
**3**	0.2	0.6	1	0.6	0.6	0.2	−0.2	0.2	0.2	−0.2	−0.6
**4**	0.6	0.2	0.6	1	0.2	0.6	0.2	−0.2	−0.2	0.2	−0.2
**5**	−0.2	0.2	0.6	0.2	1	0.6	0.2	0.6	0.6	0.2	−0.2
**6**	0.2	−0.2	0.2	0.6	0.6	1	0.6	0.2	0.2	0.6	0.2
**7**	0.6	0.2	−0.2	0.2	0.2	0.6	1	0.6	−0.2	0.2	0.6
**8**	0.2	0.6	0.2	−0.2	0.6	0.2	0.6	1	0.2	−0.2	0.2
**9**	−0.6	−0.2	0.2	−0.2	0.6	0.2	−0.2	0.2	1	0.6	0.2
**10**	−0.2	−0.6	−0.2	0.2	0.2	0.6	0.2	−0.2	0.6	1	0.6
**11**	0.2	−0.2	−0.6	−0.2	−0.2	0.2	0.6	0.2	0.2	0.6	1

We considered both positive and negative correlations among traits. There were strong positive correlations between pairs of traits such as traits 5 and 9, traits 6 and 10, and traits 7 and 11. There were also strong negative correlations between pairs of traits such as traits 1 and 9, traits 2 and 10, and traits 3 and 11. We picked some representative traits having a wide range of pairwise correlations to test the performance of the statistical methods. The selected traits included traits 1, 2, 6, 9, and 10. We focus on traits with differing correlations, positive and negative, especially with correlation 0.6 between traits 1 and 2, 0.2 between traits 1 and 6, −0.6 between traits 1 and 9, and −0.2 between traits 1 and 10.

Next, we sampled a subset of individuals from the large population, using the same sampling scheme that was used to select participants for the CHARGE targeted sequencing project. We first selected a random cohort with 504 individuals. We then sampled extremes for each of 11 traits, with participants in the extreme for one trait not eligible for selection for other traits. We chose the top 50 unselected individuals at the extremes for each of 10 traits, and then chose the top 92 to mimic one trait in FHS that had more individuals in the extreme. All individuals, regardless of selection, are analyzed using continuous traits in our case-cohort design.

We randomly assigned the generated phenotypes to genotypes for type I error tests, and denote them as *y*_0_ under the null hypothesis. We generated phenotypes for our power evaluation using the equation

1 $$ {y}_p={y}_0+{\displaystyle {\sum}_{j=1}^P{\beta}_j{G}_j,} $$

where ${\displaystyle \sum_{j=1}^P{\beta}_j{G}_j}$ indicates the additional power generated from *P* causal SNPs (coefficient *β*_*j*_ for SNP *G*_*j*_ with *j* = 1,…*P*) and *y*_0_ is generated under the null hypothesis. We randomly selected a portion of rare variants with MAF < 1% as causal variants, and the effect sizes for the causal variants were calculated by 0.4*|log10(MAF)|, following the approach of Wu et al. [13]. Power will increase with the larger the number of causal variants in this aggregate sum and the larger their effect sizes.

We selected causal variants by including all potentially functional variants as annotated by [18], while avoiding a low total number of causal variants in a region. Previous studies have used simulated genotype data and selected a low percentage of causal variants. We, however, used real targeted CHARGE sequencing genotypes, for which we can also obtain some known genetic information to aid in selection. Among SNPs with MAF < 1%, we selected all non-synonymous, stop-gain (non-sense) mutation, and splicing SNPs, because such SNPs have a higher chance to be causal, and we called them high risk variants. The number of such high risk variants for each of the 77 targeted regions in the CHARGE sequencing project varies from 0 to 81. For regions with a low number of causal variants, we selected additional causal variants using the following rules.

1. When the total number of variants for a region was low and less than 10, we selected all variants as causal regardless of whether they are high risk or not. We had 2 such regions.
2. When the number of high risk variants in a region was low and less than 5 and the total number of variants was between 10 and 100, we randomly selected an additional 50% of the variants as causal. We had 22 such regions.
3. When the number of high risk variants in a region was low and less than 5 but the total number was greater than 100, we selected an additional 5% of the non-high risk variants as causal.
4. When the number of high risk variants in a region was greater than 5, we chose all of them as causal.
5. We assigned causal variants to have the same direction of genetic effects for phenotypes using rules 1–4. We also assigned a second set of causal variants to have bi-directional effects on phenotypes using rules 1–4 by setting the first half to have positive effects and the second half to have negative effects on the phenotypes.

Note that rules 2–4 ensure that the number of causal variants in a region is 5 or more. However, removal of variants with a high missing rate (>10%) results in several regions having the number of causal variants < 5. These regions are 1, 9, 12, 18, 39 that have 4 causal variants and region 45 that has 3 causal variants.

### Statistical methods description

We chose several representative analysis methods for aggregate tests to compare, including an unweighted burden test [16], weighting of variants by a function of MAF (similar to Madsen and Browning [15], referred to “MB”), SKAT [13], and Score-Seq [14]. Let *G*_*ij*_ denote the genotype of the *j*th variant for the *i*th person with values of 0, 1, or 2 according to the number of rare alleles for variant *j*, where *i* = 1, 2, …, *n* and *j* = 1, 2, … *P*. Let *Y*_*i*_ denote the trait, and *Z*_*ik*_ the *k* th covariate for participant *i*, where *k* = 1, … *M*. We present methods for quantitative traits, but they can readily be extended to dichotomous traits.

1. **Unweighted burden test statistic (T1-Count)** [16]

For each participant, a new variable is defined that counts the number of variants (0/1/2/3…) with MAF < 1% in a targeted region where that person carries at least 1 rare allele. Association analysis with this new variable (T1-count) and a trait is carried out using linear (for a quantitative trait) or logistic (for a dichotomous trait) regression.

2. **Weighting of Variants by a function of MAF** (similar to Madsen & Browning [15] for binary traits and Xing et al. [1] for continuous traits; labeled MB in this paper)

For each person, a statistic is computed that is a weighted count of that person’s rare alleles within a targeted, using weights based on the MAF averaged over the three studies of the CHARGE targeted sequencing-project. The approach gives more weight to rarer variants. We restricted our tests to rare variants with MAF < 1%. For a targeted region, the weighted genotype score is

2 $$ {S}_i={\displaystyle \sum_{j=1}^P}\frac{G_{ij}}{{\hat{w}}_j}, $$

where ${\widehat{w}}_j=\sqrt{n{\widehat{p}}_j\left(1-{\widehat{p}}_j\right)},$ and ${\widehat{p}}_j=$ estimate of the MAF of variant *j*. Association with this genotype score and the trait of interest can be evaluated using linear or logistic regression.

3. **SKAT statistics** [13]

The Sequence Kernel Association Test (SKAT) assumes the model

3 $$ {Y}_i={\alpha}_0+{\displaystyle \sum_{k=1}^M}{\alpha}_k{Z}_{ik}+{\displaystyle \sum_{j=1}^P}{\beta}_j{G}_{ij}+{\epsilon}_i, $$

where *α*_0_, *α*_*k*_ and *β*_*j*_ are regression parameters. SKAT is a general approach and uses weights computed from the data. For our purposes in testing the null hypothesis H_0_: **β =** 0, SKAT takes a simple form, where **β** is the vector of all *β*_*j*_*s*. For a given set of weights, the score test can be expressed as

4 $$ Q = {\displaystyle \sum_{j=1}^P}{w}_j{S}_j^2, $$

where ${S}_j={\displaystyle \sum_{i=1}^n}{G}_{ij}\left({y}_i-{\widehat{\mu}}_{i0}\right),\ {\widehat{\mu}}_{i0}.$ is the predicted value of *y*_*i*_ from the model when there are no genotypes in the model. We used the Beta distribution for the weights, *w*_*j*_ ~ *Beta*(*a*_1_, *a*_2_), with the default parameters *a*_1_ = 1 d *a*_2_ = 25 as suggested by Wu et al. [13]. Association between the trait of interest and the rare variants can be evaluated using the score test, and its significance is computed analytically using a mixture of chi-square distributions.

4. **Score-Seq statistics** [14]

We can relate *Y*_*i*_ to *G*_*i*_ and *Z*_*i*_ using the following linear regression model,

Y_i_ = *τS*_*i*_ + *γ*^*T*^*Z*_*i*_ + *ϵ*_*i*_, where *ϵ*_*i*_ ~ *N*(0, *σ*^2^).

Here *S*_*i*_ = *ξ*^*T*^*G*_*i*_, a scalar from the product of a weighted linear combination of *G*_*i*1_, …, *G*_*iP*_ with weights of *ξ*_*j*_. *ξ* = (*ξ*_1_, …, *ξ*_*P*_)^*T*^ is a *P* × 1 vector, *ξ* = *β*/*τ* and *τ* is a scalar constant, and *β* is a vector of coefficients for *G*_*i*_ as defined in Equation (3) .

The score statistic and its variance are

$$ U={\displaystyle \sum_{i=1}^n}\left({Y}_i-\hat{\gamma^T}{Z}_i\right){S}_i $$

and

$$ V=\hat{\sigma^2}\left\{{\displaystyle \sum_{i=1}^n}{S}_i^2-{\left({\displaystyle \sum_{i=1}^n}{S}_i{Z}_i\right)}^T{\left({\displaystyle \sum_{i=1}^n}{Z}_i{Z^T}_i\right)}^{-1}\left({\displaystyle \sum_{i=1}^n}{S}_i{Z}_i\right)\right\}, $$

where

$$ \widehat{\gamma}={\left({\displaystyle \sum_{i=1}^n}{Z}_i{Z}_i^T\right)}^{-1}{\displaystyle \sum_{i=1}^n}{Y}_i{Z}_i, $$

and

$$ \hat{\sigma^2}={n}^{-1}{\displaystyle \sum_{i=1}^n}{\left({Y}_i-{\widehat{\gamma}}^T{Z}_i\right)}^2. $$

der *H*_0_, the test statistic *T* = *U*/*V*^1/2^ has an asymptotic standard normal distribution. Lin et al. [14] also provides permutation-type tests for more accurate p values. We implemented permutation-type tests in this article.

## Results and discussion

We applied the four methods to the simulated data for each targeted region using targeted sequence genotype data from 1096 FHS participants. We evaluated type I error and power with 10,000 replicates, at significance levels of *α* = 0.001, 0.01, and 0.05. We restricted our analyses to genetic variants with MAF < 1%. We evaluate the statistical performance of the four approaches under a case-cohort design to provide some guidelines for studies with case-cohort designs.

### Type I error

**Figure 1:**
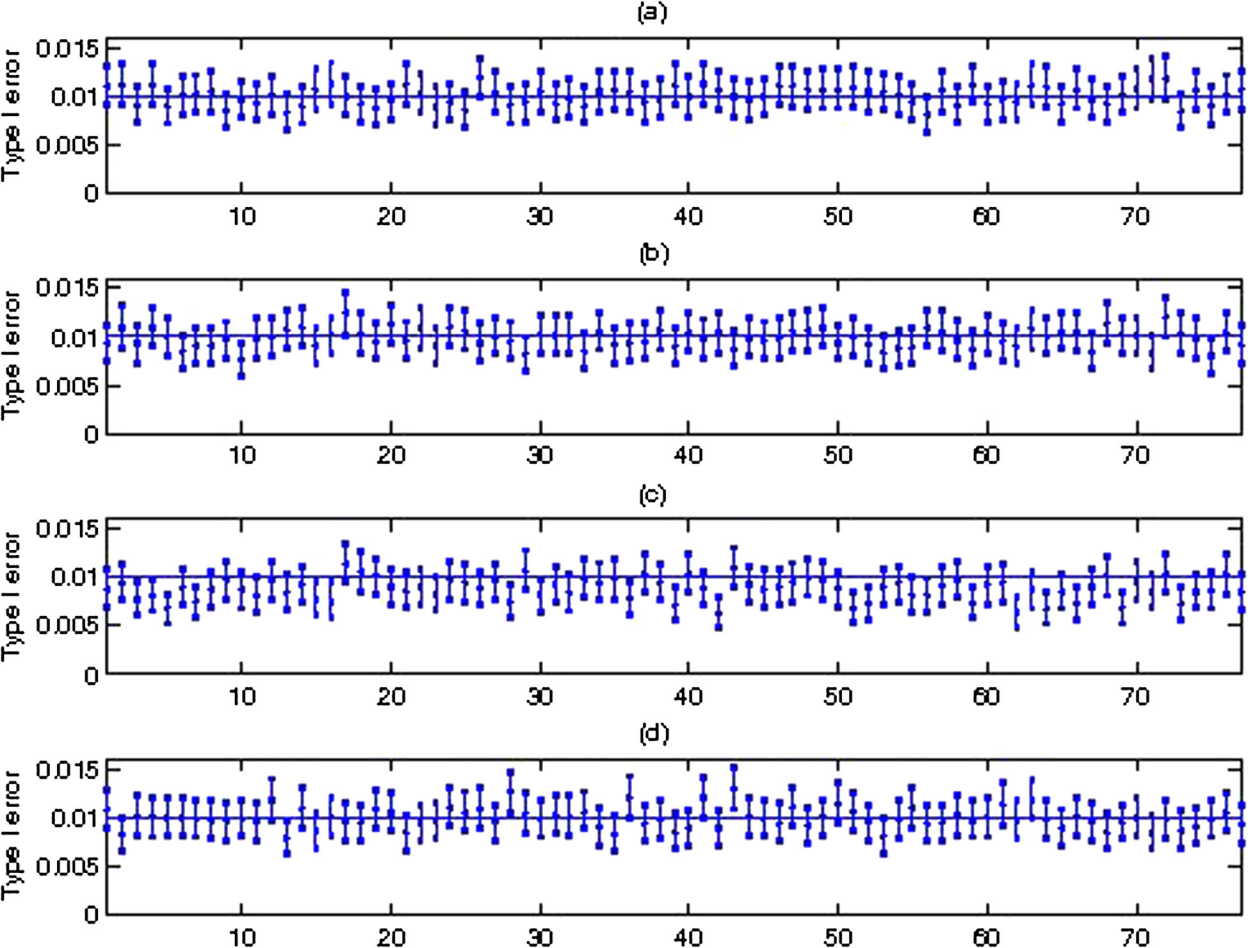
**Comparison of Type I Error for trait 1 with alpha = 0.01. (a)**. Type I error for T1. **(b)**. Type I error for Madson and Browning, 2009. **(c)**. Type I error for SKAT [13]. **(d)**. Type I error for Score-Seq [14].

Type I error, estimated by the number of rejections divided by the number of replicates (10,000), for the 77 regions for trait 1 is presented in Figure 1 for *α* = 0.01. Assuming the number of rejections follows a binomial distribution, we calculated the 95% confidence interval for the type I error, and its bounds are indicated as the two ends for each region in Figure 1. The horizontal solid line indicates the nominal level of *α* = 0.01. When the nominal level is within the 95% CI of type I error, we consider type I error to be properly controlled. We use numbers to indicate regions for simplicity of presentation. The mapping from region numbers to gene names and their chromosomes and positions are given in Additional file 1: Table S1 in the supplementary file.

From Figure 1 (a) for T1, we observe that the nominal significance level of 0.01 is within the 95% CI for most targeted regions. There are a few regions where the type I error is slightly inflated and the lower bound of the 95% CI is close to the nominal level, such as regions 26, 71 and 72. From Figure 1 (b) for MB, the type I error is also within the 95% CI for most targeted regions, with only a few regions having the lower bound of 95% CI close to the nominal level. From Figure 1 (c) for Score-Seq, the nominal level for most regions is within the 95% CI, with a few regions having a lower bound above the nominal level. From Figure 1 (d) for SKAT, no regions have type I error above the nominal level and the type I error tends to be conservative so that there are some regions with the upper bound of the 95% CI below the nominal level. As a result, SKAT has some regions with better controlled type I error than other methods such as region 24 compared to Score-Seq, although SKAT has more regions with conservative type I error. Regions 13 and 73 tend to have type I error close to or lower than the nominal level in T1, Score-Seq and SKAT. A closer look at them indicates that region 13 has 34 variants but region 73 has a large number of 257 variants with MAF < 1%. Overall, no methods have regions with 95% CIs above the nominal level except that SKAT has a few regions with the upper bound of 95% CI lower than the nominal level.

Type I error is also mostly under control when the nominal level alpha is larger at 0.05 or smaller at 0.001 (Additional file 1: Figure S1 in the Supplement). The overall control of type I error across methods is consistent with previous reports [13-15,19]. Hence, although the case-cohort study design could induce biases by including extremes when using existing statistical methods, the type I error remains under control when applying these approaches to a case-cohort design.

**Figure 2:**
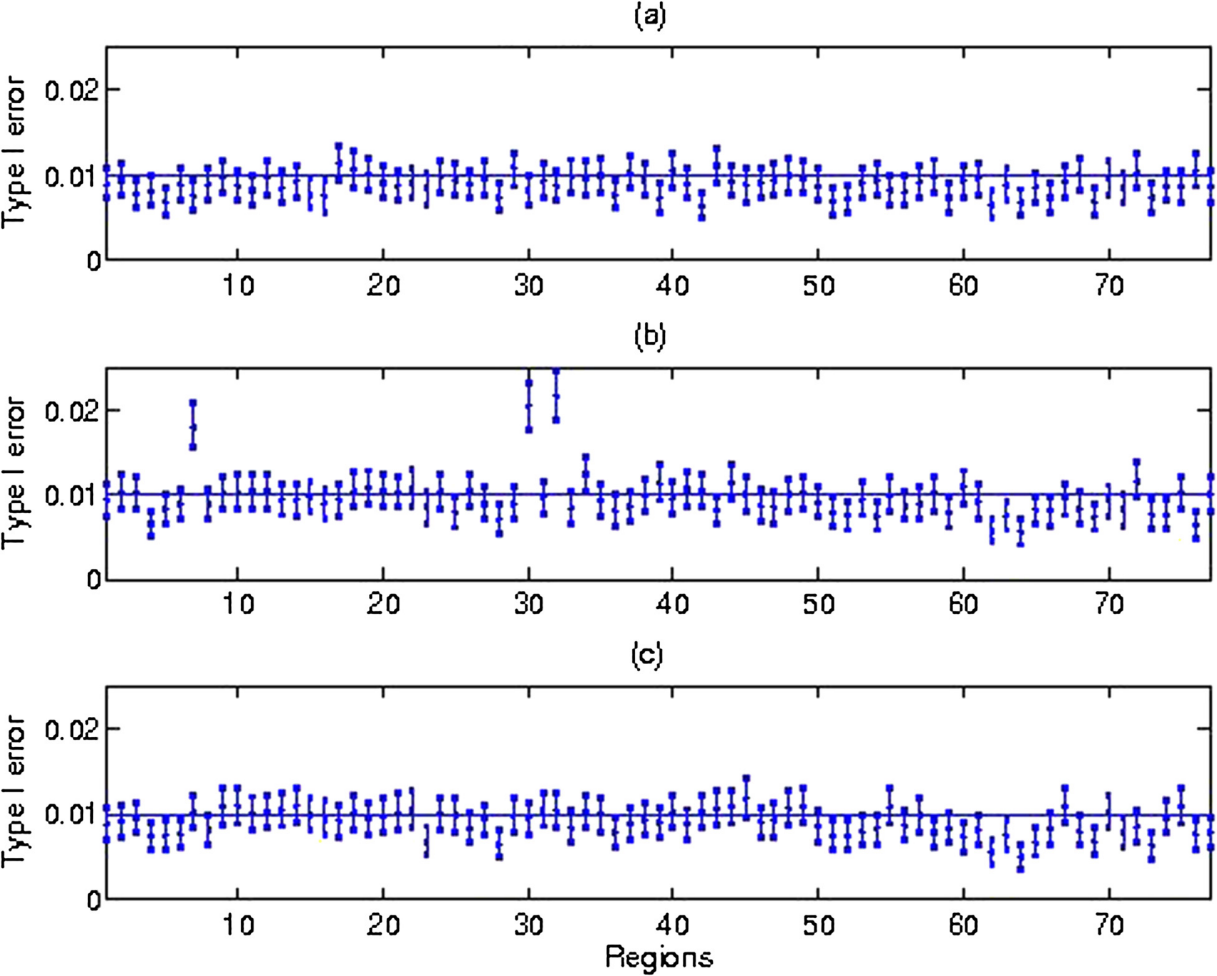
**Type I Error for SKAT over different traits when alpha = 0.01. (a)**. Type I error for trait 1. **(b)**. Type I error for trait 6. **(c)** Type I error for trait 10.

Next, we examine the variation in type I error over traits that are correlated using SKAT (Figure 2 for alpha = 0.01). We omit traits 2 and 9 because their results are similar to traits 1 and 10. Type I error over the different traits is similar and well controlled, except for three regions for trait 6 (regions 7, 30 and 32) with inflated type I error. Further investigation indicates that region 7 has 194 variants, region 30 has 9 variants, and region 32 has 6 variants with MAF < 1%. The smaller number of variants for regions 30 and 32 may increase the risk of having elevated type I error for a region, because we treated each targeted region as a unit to jointly analyze rare variants regardless of the length of a region. However, there are other regions that have fewer variants with MAF < 1% such as region 40 with 3 variants and region 9 with 4 variants, but these two regions have appropriate type I error. More summary statistics, such as the number of variants in each region, can be found from Additional file 1: Table S1 in the Supplement. We also examined type I error over traits when alpha = 0.001 and 0.05 (Additional file 1: Figure S2 in the Supplement). When alpha is smaller with a value of 0.001 or larger with a value of 0.05, the type I error for all traits tends to be conservative, except for the three regions for trait 6. The type I error for the three regions for trait 6 tend to be smaller when alpha is smaller with value of 0.001, but tend to be larger when alpha is large with value of 0.05.

### Power

**Figure 3:**
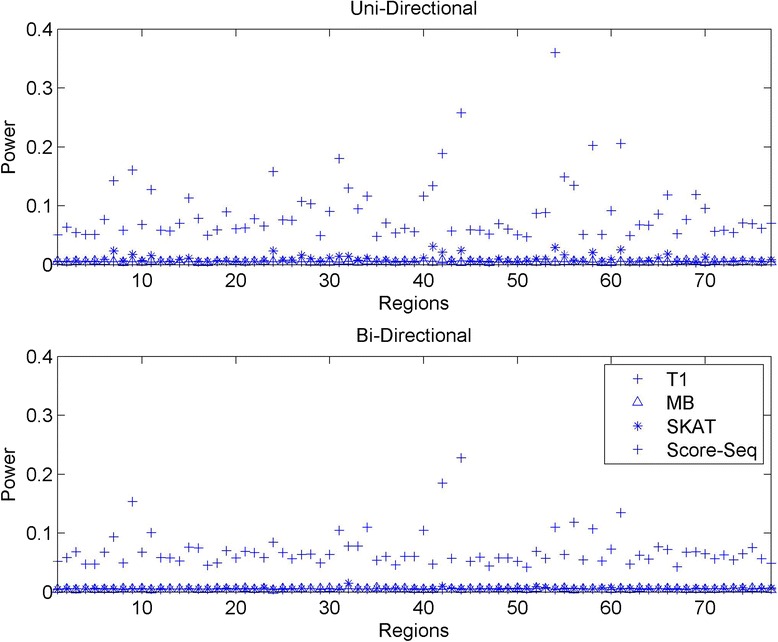
**Power for trait 1 when alpha = 0.01.**

We calculated power for the same four methods, T1, MB, SKAT and Score-Seq, and the powerfor all regions is presented in Figure 3 when all causal variants have the same directionality (the upper half of Figure 3) and when 50% of causal variants have positive effect and another 50% negative effect (the lower half of Figure 3) with alpha = 0.05. We present power multiplied by the alpha/type I error for a fair comparison across methods, with alpha as the nominal significant level. From the upper half of Figure 3, we observe that the power in our sample for 1096 individuals is generally low for detecting association with rare variants when MAF < 1%. This result is expected, because the number of simulated causal variants is generally low for most regions, varying from 4 to 60, while our sample size is modest. Generally, the power for Score-Seq tends to be higher.

We explored different characteristics of regions to investigate the possible explanations for the difference in power over regions. The characteristics included the total number of causal variants, the total allele count, and the proportion of the variance explained by the rare variants (R^2^). The summary is in Additional file 1: Table S1 of the Supplement. The power for T1 and MB are consistently low across all regions regardless of characteristics, no matter whether the genetic effects are in the same or in opposite direction.

When the number of causal variants for a region was large, the power tended to be high for Score-Seq and SKAT (Additional file 1: Figure S3 (a) of the Supplement), particularly for Score-Seq, which is consistent with the observation from [14]. For example, regions 44 and 54 have 51 and 60 causal variants, and the power of Score-Seq was 0.258 and 0.360, respectively. When the number of causal variants was modest and greater than 10, the power for Score-Seq was lower, varying from 0.05 to 0.2. When the number of causal variants was low and less than 10, the power was even lower.

We also evaluated relevant measures, total allele count and total number of variants. The total allele count was the total number of the minor alleles in all causal variants in a region. If a region had a higher total allele count, the power tended to be high (Additional file 1: Figure S3 (c) of the Supplement). However, not all regions that had higher total allele count had higher power. For example, region 73 had a total allele count of 31, but the power was only 0.006 for SKAT and was 0.055 for Score-Seq when genetic effects.

**Figure 4:**
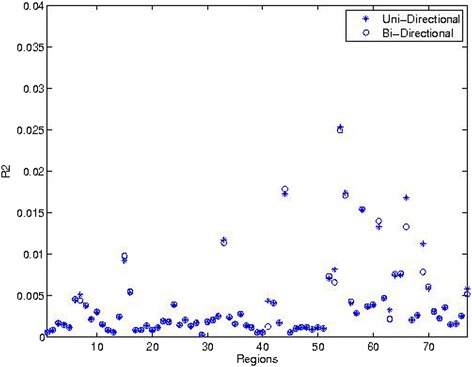
***R*** ^**2**^ **over target regions.**

We further investigated the variation in power across the regions, using the proportion of variance explained by the variants ${R}^2=\frac{var\left({\displaystyle {\sum}_{p=1}^P}{\beta}_p{G}_p\right)}{var\left({y}_p\right)}$ for each region, where ${\displaystyle \sum_{p=1}^P}{\beta}_p{G}_p$ and *y*_*p*_ are from equation (1). The calculated *R*^2^ over regions is given in Figure 4, with the overall mean *R*^2^ equal to 0.0040. Looking at Figures 3 and 4, both methods tended to have larger power when *R*^2^ is larger, particularly Score-Seq with a larger increase (Additional file 1: Figure S3 (d) in the Supplement).

We investigated the power when causal variants have bi-directionality with 50% of variants having a positive effect and 50% of variants having a negative effect. The influence of relevant measures for power is plotted in Additional file 1: Figure S3 in the Supplement, when the causal effects are both uni-directional and bi-directional. The power over 77 regions is plotted in the lower half of Figure 3. The power for SKAT was low, and did not change much compared with the scenarios having the same directionality of genetic effect. The power for Score-Seq however decreased. The other characteristics of the targeted regions including the total number of causal variants and the total allele count did not change the power for SKAT much, compared to the power evaluated when all rare alleles shared the same directionality. This result is expected, as the power for SKAT is closely related to R^2^ and is robust to directionality (Figure 4). Further observation indicates that the power for alpha = 0.01 tended to be lower (Additional file 1: Figure S4 in the Supplement). Other factors that may influence the power include sample size and weighting schemes/distributions of effects [14,17].

## Conclusion

Many statistical methods have been developed in recent years to evaluate the risk conferred by rare variants in human complex diseases. However, no statistical method has considered the case-cohort design. We evaluated several approaches to access the association between groups of rare variants and a complex quantitative trait, because most FHS traits in the targeted sequencing project were quantitative. We aimed to evaluate several representative statistical methods instead of a comprehensive evaluation of all existing methods and compared their performance on our CHARGE targeted sequencing data. Our work contributes in that we are the first to evaluate both type I error and power using a case-cohort design of observed targeted CHARGE sequencing data. Seventy-seven targeted regions represent a wide range of genotypic characteristics from real sequencing data, and we evaluated the performance using correlated complex phenotypes by mimicking the sampling scheme used in CHARGE targeted sequencing project.

Type I error in the case-cohort design of CHARGE targeted sequencing data was mostly under control, although type I error for SKAT tended to be conservative. We tested the type I error over correlated traits using SKAT. Most regions for most traits had appropriate type 1 error. These results suggested that correlated complex phenotypes in a case-cohort design may influence the behavior of type I error but not substantially. Power was generally low in our studies no matter whether the effects of causal variants have the same directionality or bi-directionality, consistent with observations in previous studies [13,14,17]. As SKAT tended to have somewhat conservative type I error, power should be evaluated carefully, considering the type I error for each method in a particular region.

We examined different characteristics of targeted regions to explore possible explanations for the difference in power across regions in the case-cohort design based on CHARGE targeted sequencing data. The characteristics that we examined included the total number of causal variants, the total allele count, the proportion of variance explained by the causal variants (R^2^), the significance level and directionality. Power for Score-Seq tended to be higher, when the total number of causal variants and the total allele count were larger. Score-Seq tended to have higher power when R^2^ is larger. Bi-directionality does not seem to influence power much for SKAT, but lowers the power for Score-Seq. Other characteristics were also investigated in prior reports to determine the influence on power of characteristics such as the ratio of causal variants to total number of variants, effect sizes and sample sizes [13,14,17]. The proportion of the total number of causal variants among the total number of variants in a region is often used when the regions have a similar number of variants [13]. Our studies used effect sizes of the form 0.4*|log10(MAF)| [13] to generate larger effect sizes for rarer variants. Our Targeted Sequencing study in FHS had a fixed sample size of 1096, and hence we did not examine the influence of varying sample sizes. Although our results for power are limited to this sample size, we expect that comparisons across methods will be similar. Our work could also be extended to exome sequencing by applying tests to all variants within the exome of a gene, or within subsets of exons of a gene. Future improvements for statistical methods and a better understanding of the underlying genetic structure may aid in evaluating rare variant association studies.

### Availability of supporting data

The data set supporting the results of this article is available in the dbGAP repository, http://www.ncbi.nlm.nih.gov/projects/gap/cgi-bin/study.cgi?study_id=phs000651.v3.p8.

## Additional file

Additional file 1:
**The summary table of target regions and the additional figures.**
