# Supplementary material for: Performance of statistical methods on CHARGE targeted sequencing data
Source: BMC Genet. 2014 Oct 3;15:104. doi: 10.1186/s12863-014-0104-9 (PMC4197341; doi:10.1186/s12863-014-0104-9)
Supplement: Additional file 1: — The summary table of target regions and the additional figures. [file 12863_2014_104_MOESM1_ESM.docx]

**Table 1: Summary of target regions.**

The total # variants represents the total number of variants in a region with MAF < 1%; total # alleles represents the total number of alleles in a region; total # causal variants represents the total number of causal variants in a region; R2 represents the proportion of variance explained by the casual variants with the same directionality; Skat-One represents power of SKAT with the same directionality of casual variants; Score-One represents power of Score-Seq with the same directionality of causal variants; the columns of R2-Bi, Skat-Bi and Score-Bi are their corresponding measures with bi-directionality. The power is calculated when alpha = 0.05.

| **Region #** | **Chromosome_Start Position_End Position** | **Gene Name** | **Total # variants** | **Total # Alleles** | **Total # Causal Variants** | **R2-1** | **T1-1** | **MB-1** | **SKAT-1** | **Score-Seq-1** | **R2-2** | **T1-2** | **MB-2** | **SKAT-2** | **Score-Seq-2** |
| --- | --- | --- | --- | --- | --- | --- | --- | --- | --- | --- | --- | --- | --- | --- | --- |
| 1 | chr1_152641684_152709758 | IL6R | 402 | 4 | 4 | 0.001 | 0.005 | 0.006 | 0.005 | 0.050 | 0.001 | 0.005 | 0.004 | 0.005 | 0.052 |
| 2 | chr1_168853417_168975265 | PRRX1 | 60 | 5 | 5 | 0.001 | 0.005 | 0.004 | 0.005 | 0.063 | 0.001 | 0.005 | 0.005 | 0.005 | 0.058 |
| 3 | chr1_65830704_65880672 | LEPR | 301 | 19 | 6 | 0.002 | 0.004 | 0.006 | 0.006 | 0.054 | 0.002 | 0.006 | 0.004 | 0.006 | 0.068 |
| 4 | chr1_68336635_68480941 | WLS | 1048 | 10 | 6 | 0.002 | 0.006 | 0.005 | 0.005 | 0.051 | 0.002 | 0.005 | 0.005 | 0.005 | 0.047 |
| 5 | chr10_104579177_104619322 | CYP17A1 | 193 | 8 | 8 | 0.001 | 0.005 | 0.006 | 0.005 | 0.051 | 0.001 | 0.005 | 0.004 | 0.006 | 0.047 |
| 6 | chr10_18468647_18871794 | CACNB2 | 274 | 37 | 7 | 0.005 | 0.005 | 0.005 | 0.009 | 0.077 | 0.005 | 0.005 | 0.004 | 0.006 | 0.068 |
| 7 | chr10_70698661_70832743 | HK1 | 194 | 29 | 9 | 0.005 | 0.005 | 0.005 | 0.023 | 0.143 | 0.004 | 0.005 | 0.005 | 0.005 | 0.093 |
| 8 | chr11_16764687_16993639 | PLEKHA7 | 253 | 20 | 11 | 0.004 | 0.006 | 0.004 | 0.006 | 0.058 | 0.004 | 0.005 | 0.005 | 0.005 | 0.050 |
| 9 | chr11_46308577_46364989 | DGKZ | 4 | 16 | 4 | 0.002 | 0.005 | 0.005 | 0.017 | 0.161 | 0.002 | 0.005 | 0.005 | 0.005 | 0.154 |
| 10 | chr11_46370216_46583755 | AMBRA1 | 48 | 22 | 6 | 0.003 | 0.005 | 0.004 | 0.007 | 0.068 | 0.003 | 0.005 | 0.006 | 0.005 | 0.067 |
| 11 | chr11_46594433_46653784 | ATG13 | 9 | 22 | 5 | 0.002 | 0.005 | 0.006 | 0.015 | 0.128 | 0.002 | 0.005 | 0.004 | 0.005 | 0.101 |
| 12 | chr11_46655000_46678200 | ARHGAP1 | 53 | 4 | 4 | 0.001 | 0.005 | 0.005 | 0.005 | 0.058 | 0.001 | 0.004 | 0.005 | 0.005 | 0.058 |
| 13 | chr11_46678300_46690000 | ZNF408 | 34 | 5 | 5 | 0.001 | 0.006 | 0.006 | 0.005 | 0.057 | 0.001 | 0.005 | 0.005 | 0.005 | 0.058 |
| 14 | chr11_46695000_46720000 | F2 | 43 | 23 | 8 | 0.002 | 0.004 | 0.005 | 0.009 | 0.070 | 0.003 | 0.005 | 0.005 | 0.005 | 0.053 |
| 15 | chr11_46720500_46832766 | CKAP5 | 143 | 71 | 14 | 0.009 | 0.005 | 0.005 | 0.011 | 0.113 | 0.010 | 0.005 | 0.005 | 0.006 | 0.076 |
| 16 | chr11_46832307_46907610 | LRP4 | 150 | 29 | 20 | 0.005 | 0.005 | 0.004 | 0.006 | 0.079 | 0.006 | 0.005 | 0.004 | 0.005 | 0.075 |
| 17 | chr11_46908492_47145542 | C11ORF49 | 87 | 5 | 5 | 0.001 | 0.005 | 0.004 | 0.005 | 0.050 | 0.001 | 0.005 | 0.004 | 0.005 | 0.046 |
| 18 | chr11_47181042_47217500 | DDB | 26 | 4 | 4 | 0.001 | 0.007 | 0.005 | 0.005 | 0.059 | 0.001 | 0.005 | 0.005 | 0.005 | 0.049 |
| 19 | chr11_47217501_47227099 | ACP2 | 9 | 22 | 5 | 0.001 | 0.005 | 0.005 | 0.006 | 0.090 | 0.001 | 0.005 | 0.005 | 0.006 | 0.070 |
| 20 | chr11_47227100_47247262 | NR1H3 | 20 | 5 | 5 | 0.001 | 0.004 | 0.006 | 0.006 | 0.061 | 0.001 | 0.005 | 0.005 | 0.006 | 0.057 |
| 21 | chr11_47246227_47309761 | MADD | 76 | 8 | 6 | 0.001 | 0.005 | 0.004 | 0.005 | 0.062 | 0.001 | 0.005 | 0.007 | 0.005 | 0.069 |
| 22 | chr11_47309354_47331966 | MYBPC3 | 27 | 10 | 7 | 0.002 | 0.005 | 0.005 | 0.005 | 0.078 | 0.002 | 0.005 | 0.004 | 0.005 | 0.067 |
| 23 | chr11_47332576_47379797 | SPI1 | 61 | 12 | 5 | 0.002 | 0.005 | 0.006 | 0.007 | 0.065 | 0.002 | 0.005 | 0.006 | 0.007 | 0.058 |
| 24 | chr11_47382745_47395672 | SLC39A13 | 14 | 22 | 5 | 0.004 | 0.005 | 0.005 | 0.023 | 0.158 | 0.004 | 0.004 | 0.003 | 0.005 | 0.084 |
| 25 | chr11_47395530_47407907 | PSMC3 | 32 | 12 | 5 | 0.001 | 0.006 | 0.005 | 0.008 | 0.076 | 0.001 | 0.006 | 0.005 | 0.004 | 0.067 |
| 26 | chr11_47409682_47437697 | RAPSN | 28 | 12 | 6 | 0.002 | 0.004 | 0.005 | 0.008 | 0.075 | 0.002 | 0.006 | 0.005 | 0.005 | 0.056 |
| 27 | chr11_47440101_47539393 | CELF1 | 25 | 22 | 5 | 0.001 | 0.005 | 0.005 | 0.016 | 0.107 | 0.001 | 0.006 | 0.006 | 0.006 | 0.064 |
| 28 | chr11_47555577_47564776 | NDUFS3 | 7 | 7 | 7 | 0.002 | 0.005 | 0.004 | 0.010 | 0.103 | 0.002 | 0.005 | 0.005 | 0.006 | 0.064 |
| 29 | chr11_47577579_47629391 | MTCH2 | 17 | 5 | 5 | 0.000 | 0.006 | 0.004 | 0.005 | 0.049 | 0.000 | 0.005 | 0.006 | 0.004 | 0.049 |
| 30 | chr11_47643741_47697000 | AGBL2 | 9 | 10 | 6 | 0.002 | 0.005 | 0.004 | 0.011 | 0.090 | 0.002 | 0.006 | 0.006 | 0.005 | 0.063 |
| 31 | chr11_47697500_47747749 | FNBP4 | 8 | 17 | 8 | 0.002 | 0.004 | 0.006 | 0.014 | 0.180 | 0.002 | 0.005 | 0.005 | 0.005 | 0.105 |
| 32 | chr11_47751382_47851121 | NUP160 | 6 | 13 | 7 | 0.003 | 0.006 | 0.006 | 0.014 | 0.130 | 0.003 | 0.006 | 0.005 | 0.015 | 0.078 |
| 33 | chr12_101312706_101455233 | IGF1 | 458 | 66 | 29 | 0.012 | 0.006 | 0.004 | 0.007 | 0.095 | 0.011 | 0.005 | 0.005 | 0.005 | 0.078 |
| 34 | chr12_110322484_110374300 | SH2B3 | 104 | 17 | 8 | 0.002 | 0.005 | 0.006 | 0.011 | 0.116 | 0.002 | 0.005 | 0.005 | 0.005 | 0.110 |
| 35 | chr12_110374301_110521963 | ATXN2 | 542 | 11 | 6 | 0.002 | 0.005 | 0.006 | 0.005 | 0.048 | 0.002 | 0.005 | 0.007 | 0.006 | 0.054 |
| 36 | chr12_110520750_110608293 | BRAP | 87 | 15 | 9 | 0.003 | 0.005 | 0.006 | 0.008 | 0.071 | 0.003 | 0.005 | 0.005 | 0.005 | 0.060 |
| 37 | chr12_110608294_110683564 | ACAD10 | 110 | 6 | 6 | 0.001 | 0.004 | 0.005 | 0.005 | 0.054 | 0.001 | 0.007 | 0.005 | 0.005 | 0.046 |
| 38 | chr12_110683464_110758296 | ALDH2 | 50 | 8 | 7 | 0.001 | 0.006 | 0.006 | 0.005 | 0.062 | 0.001 | 0.005 | 0.005 | 0.005 | 0.060 |
| 39 | chr12_110759690_110818343 | MAPKAPK5 | 69 | 4 | 4 | 0.001 | 0.004 | 0.005 | 0.005 | 0.056 | 0.001 | 0.004 | 0.005 | 0.006 | 0.060 |
| 40 | chr12_110820114_110824982 | ADAM1 | 3 | 7 | 5 | 0.001 | 0.006 | 0.005 | 0.011 | 0.116 | 0.001 | 0.005 | 0.004 | 0.004 | 0.104 |
| 41 | chr12_110836628_110940051 | TMEM116 | 51 | 27 | 7 | 0.004 | 0.005 | 0.005 | 0.031 | 0.134 | 0.001 | 0.006 | 0.004 | 0.005 | 0.047 |
| 42 | chr12_110939450_111036527 | NAA25 | 68 | 24 | 10 | 0.004 | 0.004 | 0.007 | 0.021 | 0.189 | 0.004 | 0.005 | 0.005 | 0.009 | 0.185 |
| 43 | chr12_111042334_111079555 | TRAFD1 | 43 | 9 | 8 | 0.002 | 0.005 | 0.005 | 0.006 | 0.057 | 0.002 | 0.007 | 0.005 | 0.005 | 0.057 |
| 44 | chr12_111080891_111237689 | C12ORF51 | 180 | 89 | 51 | 0.017 | 0.006 | 0.004 | 0.024 | 0.258 | 0.018 | 0.004 | 0.004 | 0.006 | 0.228 |
| 45 | chr12_111324467_111334297 | RPL6 | 12 | 3 | 3 | 0.001 | 0.005 | 0.005 | 0.006 | 0.059 | 0.001 | 0.005 | 0.005 | 0.005 | 0.052 |
| 46 | chr12_111338491_111436622 | PTPN11 | 35 | 6 | 6 | 0.001 | 0.005 | 0.006 | 0.006 | 0.058 | 0.001 | 0.005 | 0.006 | 0.005 | 0.059 |
| 47 | chr12_119899944_119925301 | HNF1A | 125 | 6 | 5 | 0.001 | 0.004 | 0.004 | 0.005 | 0.052 | 0.001 | 0.005 | 0.005 | 0.005 | 0.044 |
| 48 | chr12_23576398_24607747 | SOX5 | 80 | 19 | 5 | 0.001 | 0.004 | 0.005 | 0.010 | 0.069 | 0.001 | 0.004 | 0.006 | 0.005 | 0.058 |
| 49 | chr12_24611065_24629469 | C12ORF67 | 22 | 6 | 5 | 0.001 | 0.004 | 0.005 | 0.006 | 0.060 | 0.001 | 0.006 | 0.005 | 0.005 | 0.057 |
| 50 | chr12_543643_740130 | NINJ2 | 1243 | 24 | 5 | 0.001 | 0.004 | 0.006 | 0.005 | 0.050 | 0.001 | 0.005 | 0.006 | 0.006 | 0.052 |
| 51 | chr12_88504856_88616005 | ATP2B1 | 534 | 6 | 5 | 0.001 | 0.006 | 0.006 | 0.006 | 0.047 | 0.001 | 0.005 | 0.004 | 0.006 | 0.042 |
| 52 | chr13_109599195_109758700 | COL4A1 | 199 | 45 | 17 | 0.007 | 0.005 | 0.004 | 0.009 | 0.087 | 0.007 | 0.005 | 0.006 | 0.009 | 0.069 |
| 53 | chr13_109757531_109967539 | COL4A2 | 144 | 32 | 17 | 0.008 | 0.004 | 0.006 | 0.009 | 0.089 | 0.007 | 0.006 | 0.005 | 0.008 | 0.057 |
| 54 | chr16_71378464_71651135 | ZFHX3 | 157 | 141 | 60 | 0.025 | 0.005 | 0.004 | 0.029 | 0.360 | 0.025 | 0.004 | 0.005 | 0.005 | 0.110 |
| 55 | chr2_226728697_226856269 | 2q36.3 | 808 | 97 | 46 | 0.017 | 0.005 | 0.006 | 0.017 | 0.149 | 0.017 | 0.005 | 0.006 | 0.006 | 0.064 |
| 56 | chr2_227236803_227388200 | IRS1 | 629 | 44 | 14 | 0.004 | 0.007 | 0.005 | 0.005 | 0.134 | 0.004 | 0.006 | 0.004 | 0.005 | 0.118 |
| 57 | chr2_27567127_27603561 | GCKR | 203 | 18 | 11 | 0.003 | 0.005 | 0.005 | 0.006 | 0.051 | 0.003 | 0.006 | 0.005 | 0.005 | 0.054 |
| 58 | chr2_586432_677539 | TMEM18 | 716 | 75 | 39 | 0.015 | 0.005 | 0.006 | 0.021 | 0.202 | 0.015 | 0.005 | 0.005 | 0.005 | 0.107 |
| 59 | chr20_10565132_10609308 | JAG1 | 370 | 20 | 12 | 0.004 | 0.005 | 0.004 | 0.006 | 0.051 | 0.004 | 0.005 | 0.005 | 0.007 | 0.053 |
| 60 | chr3_38511667_38552500 | EXOG | 122 | 22 | 10 | 0.004 | 0.004 | 0.004 | 0.009 | 0.091 | 0.004 | 0.004 | 0.005 | 0.007 | 0.073 |
| 61 | chr3_38564457_38667267 | SCN5A | 132 | 74 | 29 | 0.013 | 0.006 | 0.005 | 0.025 | 0.206 | 0.014 | 0.005 | 0.005 | 0.005 | 0.135 |
| 62 | chr3_38694789_38811605 | SCN10A | 1016 | 23 | 19 | 0.005 | 0.005 | 0.004 | 0.005 | 0.050 | 0.005 | 0.006 | 0.005 | 0.006 | 0.047 |
| 63 | chr5_147815702_147837626 | MEF2C_1 | 231 | 10 | 10 | 0.003 | 0.004 | 0.004 | 0.006 | 0.068 | 0.002 | 0.004 | 0.006 | 0.006 | 0.062 |
| 64 | chr5_156830995_156936446 | MEF2C_2 | 798 | 36 | 13 | 0.007 | 0.005 | 0.005 | 0.007 | 0.067 | 0.008 | 0.005 | 0.005 | 0.006 | 0.056 |
| 65 | chr5_87819438_88215292 | HTR4 | 184 | 56 | 17 | 0.008 | 0.005 | 0.005 | 0.011 | 0.086 | 0.008 | 0.006 | 0.005 | 0.005 | 0.077 |
| 66 | chr5_88335639_88457219 | ADAM19 | 775 | 69 | 46 | 0.017 | 0.006 | 0.004 | 0.018 | 0.119 | 0.013 | 0.004 | 0.006 | 0.006 | 0.072 |
| 67 | chr6_118970965_118989480 | SLC17A4 | 988 | 10 | 8 | 0.002 | 0.005 | 0.006 | 0.005 | 0.052 | 0.002 | 0.006 | 0.005 | 0.005 | 0.042 |
| 68 | chr6_135322113_135417715 | PLN | 124 | 14 | 11 | 0.003 | 0.005 | 0.007 | 0.006 | 0.077 | 0.003 | 0.005 | 0.004 | 0.005 | 0.068 |
| 69 | chr6_135417716_135544145 | HBS1L | 106 | 47 | 25 | 0.011 | 0.004 | 0.006 | 0.005 | 0.119 | 0.008 | 0.005 | 0.004 | 0.006 | 0.068 |
| 70 | chr6_135544146_135582124 | 6q23.3 | 443 | 48 | 8 | 0.006 | 0.004 | 0.004 | 0.013 | 0.095 | 0.006 | 0.005 | 0.004 | 0.005 | 0.065 |
| 71 | chr6_25857845_25987550 | MYB | 67 | 18 | 9 | 0.003 | 0.004 | 0.006 | 0.005 | 0.056 | 0.003 | 0.006 | 0.004 | 0.005 | 0.057 |
| 72 | chr7_100054874_100079499 | TFR2 | 111 | 11 | 7 | 0.002 | 0.005 | 0.005 | 0.005 | 0.058 | 0.002 | 0.005 | 0.006 | 0.006 | 0.063 |
| 73 | chr7_105917292_106379327 | PIK3CG | 257 | 31 | 14 | 0.004 | 0.005 | 0.004 | 0.006 | 0.055 | 0.004 | 0.006 | 0.005 | 0.006 | 0.054 |
| 74 | chr7_115925580_115935931 | CAV2 | 30 | 8 | 7 | 0.002 | 0.006 | 0.006 | 0.007 | 0.071 | 0.002 | 0.004 | 0.006 | 0.007 | 0.065 |
| 75 | chr7_115950975_115988574 | CAV1 | 49 | 13 | 8 | 0.002 | 0.006 | 0.005 | 0.006 | 0.069 | 0.002 | 0.005 | 0.006 | 0.006 | 0.075 |
| 76 | chr7_150856537_151267715 | PRKAG2 | 352 | 24 | 6 | 0.003 | 0.005 | 0.004 | 0.005 | 0.062 | 0.003 | 0.006 | 0.006 | 0.005 | 0.056 |
| 77 | chr8_27783553_27906232 | SCARA5 | 167 | 32 | 13 | 0.006 | 0.005 | 0.005 | 0.008 | 0.070 | 0.005 | 0.005 | 0.004 | 0.007 | 0.049 |


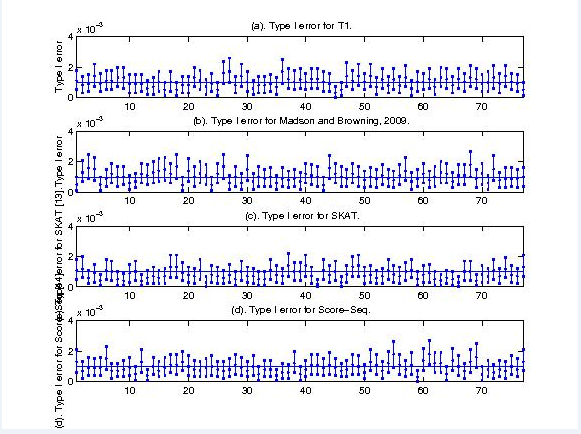


**Figure 1 (a): Type I error for four analysis methods across regions when alpha = 0.001.**


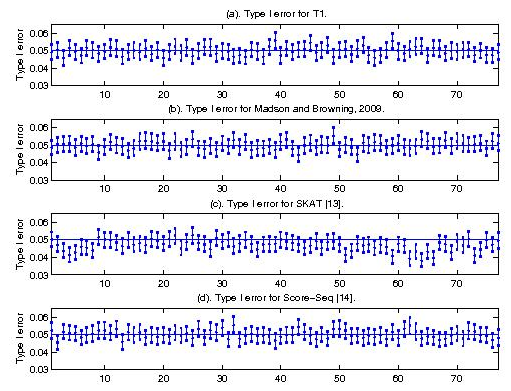


**Figure 1 (b): Type I error for four analysis methods across regions when alpha = 0.05.**


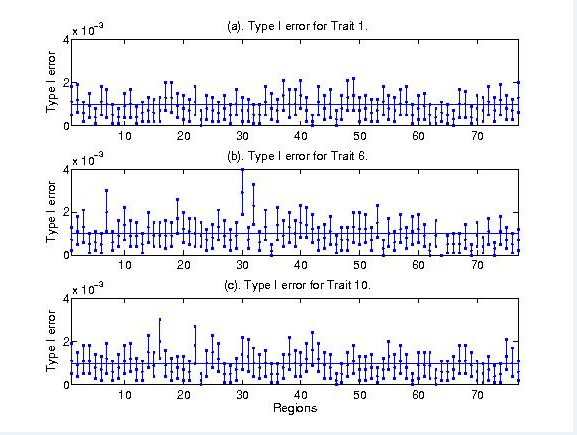


**Figure 2 (a): Type I error for three traits across regions when alpha = 0.001**

**
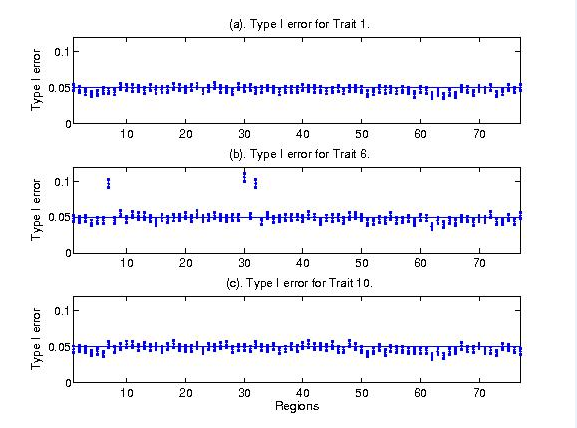
**

**Figure 2 (b): Type I error for three traits across regions when alpha = 0.05**

**
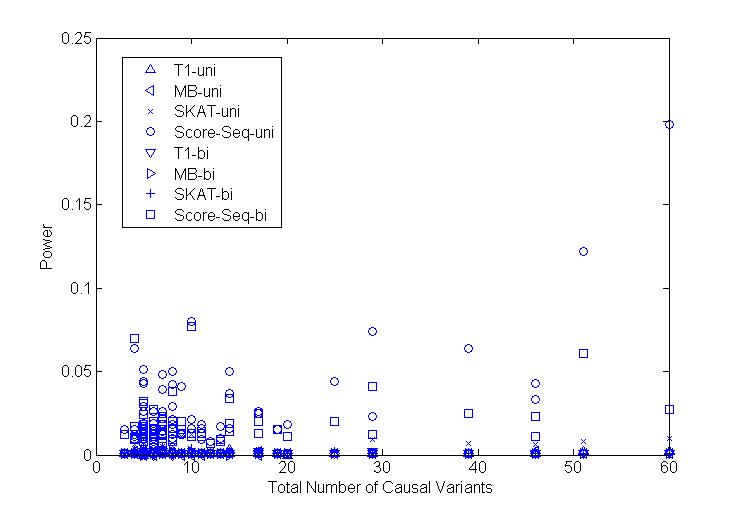
**

**Figure 3 (a): The influence of the total number of casual variants to power when alpha = 0.01.**


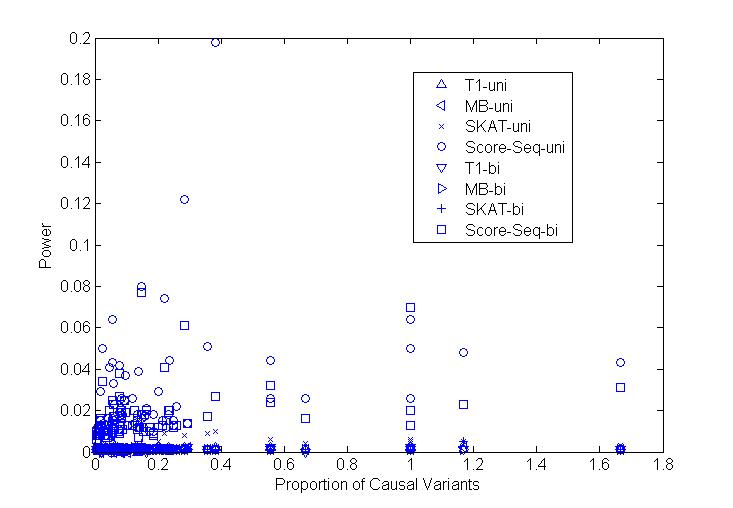


**Figure 3 (b): The influence of the percentage of casual variants to power when alpha = 0.01.**

**
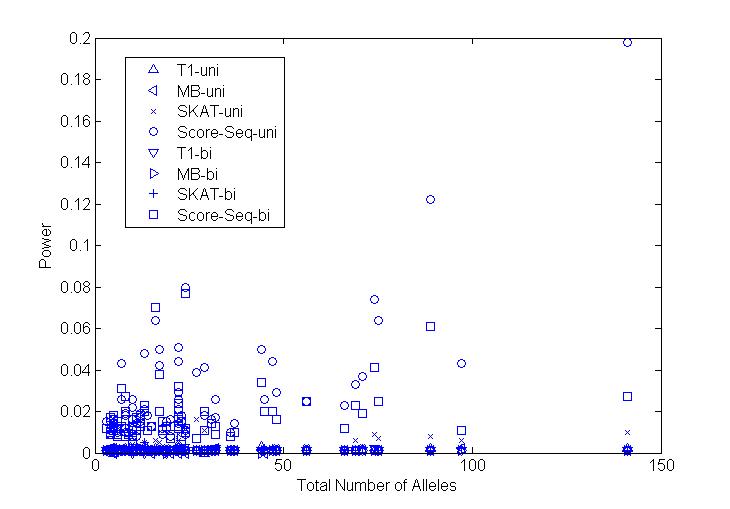
**

**Figure 3 (c): The influence of the total number of alleles to power when alpha = 0.01.**

**
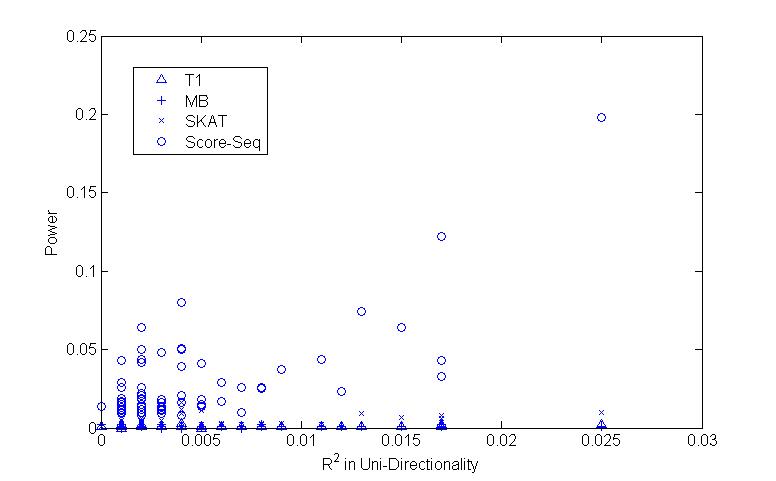
**

**Figure 3 (d): The influence of R^2^ to power when casual effects are uni-directional when alpha = 0.01.**

**
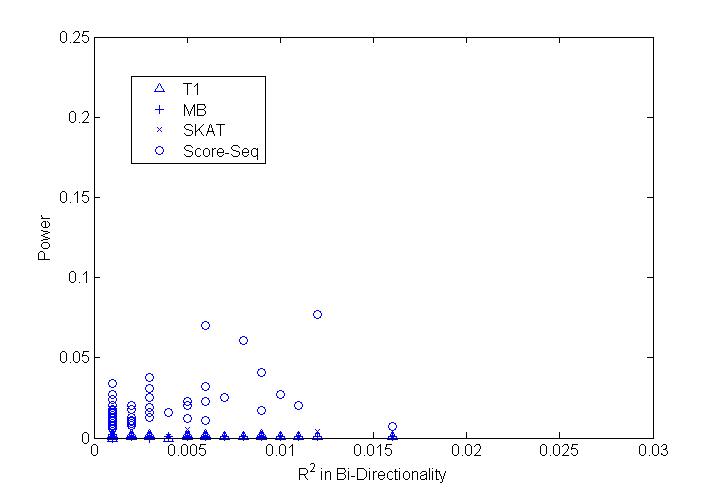
**

**Figure 3 (e): The influence of R^2^ to power when casual effects are bi-directional when alpha = 0.01.**


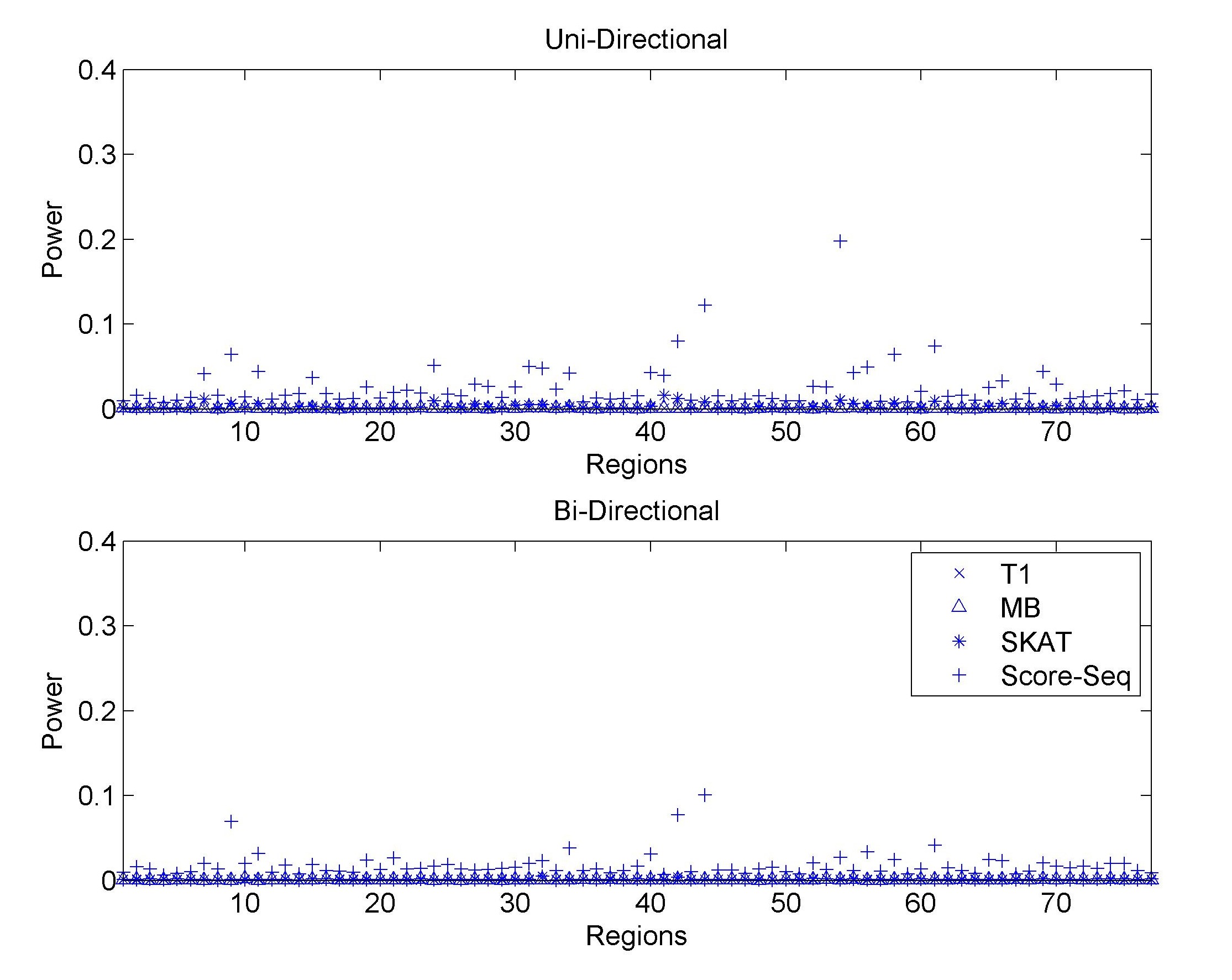


**Figure 4: Power when alpha = 0.01**
